# Gait analysis in the early stage of Parkinson’s disease with a machine learning approach

**DOI:** 10.3389/fneur.2024.1472956

**Published:** 2024-10-08

**Authors:** Wenchao Yin, Wencheng Zhu, Hong Gao, Xiaohui Niu, Chenxin Shen, Xiangmin Fan, Cui Wang

**Affiliations:** ^1^Department of Neurology, Central Hospital of Dalian University of Technology, Dalian, China; ^2^Beijing CAS-Ruiyi Information Technology Co., Ltd., Beijing, China; ^3^School of Science, Dalian Maritime University, Dalian, China; ^4^Institute of Software, Chinese Academy of Sciences, Beijing, China

**Keywords:** Parkinson’s disease, gait analysis, early-stage diagnosis, MDS-UPDRS III score, non-contact assessment, machine learning

## Abstract

**Background:**

Gait disorder is a prominent motor symptom in Parkinson’s disease (PD), objective and quantitative assessment of gait is essential for diagnosing and treating PD, particularly in its early stage.

**Methods:**

This study utilized a non-contact gait assessment system to investigate gait characteristics between individuals with PD and healthy controls, with a focus on early-stage PD. Additionally, we trained two machine learning models to differentiate early-stage PD patients from controls and to predict MDS-UPDRS III score.

**Results:**

Early-stage PD patients demonstrated reduced stride length, decreased gait speed, slower stride and swing speeds, extended turning time, and reduced cadence compared to controls. Our model, after an integrated analysis of gait parameters, accurately identified early-stage PD patients. Moreover, the model indicated that gait parameters could predict the MDS-UPDRS III score using a machine learning regression approach.

**Conclusion:**

The non-contact gait assessment system facilitates the objective and quantitative evaluation of gait disorder in PD patients, effectively distinguishing those in the early stage from healthy individuals. The system holds significant potential for the early detection of PD. It also harnesses gait parameters for a reasoned prediction of the MDS-UPDRS III score, thereby quantifying disease severity. Overall, gait assessment is a valuable method for the early identification and ongoing monitoring of PD.

## 1 Introduction

Parkinson’s disease (PD) is one of the most common neurodegenerative diseases, affecting approximately 1% of the population over 60 years of age (1). The typical motor symptoms of PD include resting tremor, bradykinesia, rigidity, postural and gait disorders. Among these, gait disorder is one of the principal symptoms in PD patients. Patients exhibit characteristic gait patterns such as reduced turning agility, short and slow steps, festinating, and freezing of gait (2, 3). Given the strong correlation between gait disorders and diminished quality of life, precise gait assessment is vital (4, 5). However, it is challenging for neurologists to assess gait in the early stage of PD (6). Early gait disorders are very subtle, and some patients even exhibit motor symptoms without conscious gait complaints (7); Additionally, the presence of short and slow steps is a common trait in the aging population (8, 9), complicating the differentiation of PD-induced gait changes from age-related alterations. Consequently, precise identification and surveillance of gait anomalies are essential for the effective treatment and prognosis of PD, particularly in its early stage.

Currently, the clinical assessments of gait in PD patients rely on traditional scales, such as the Section III of the modified movement disorder society version of the unified Parkinson’s disease rating scale (MDS-UPDRS III), the Timed-Up and Go test, and the Freezing of Gait Questionnaire, and so on (10). However, these scales depend on the subjective assessments of clinical physicians and have the limitations of being semi-quantitative, time-consuming, and potentially leading to inconsistent and imprecise results. In recent years, along with the rapid advancement of science and technology, a variety of objective and quantitative gait assessment techniques have gradually matured, propelling PD gait research into a new stage (11), such as multi-camera motion capture systems, wearable sensors, and pressure-sensitive insoles. However, the existing methods also have various shortcomings. For instance, multi-camera motion capture systems offer the highest capture accuracy and are considered as the golden standard in clinical gait analysis (11), yet they are costly and demand a large space, making them difficult to popularize currently. Wearable sensors, while portable, still face several challenges, such as discomfort during wear, data synchronization, and noise contamination. In summary, although new technologies show great potential in gait assessment in PD, they still need further improvement and optimization in terms of popularization and clinical application (12). In order to provide more refined and convenient gait monitoring methods for PD patients, future research should focus on enhancing the universality of the technology, reducing costs, improving user experience, and maintaining the accuracy and reliability of the data in the same time.

Based on the current challenges faced by PD gait assessments, we have utilized a non-contact gait assessment system (ReadyGo, Beijing CAS-Ruiyi Information Technology Co., Ltd.) (13), in order to overcome the limitations of traditional evaluation methods. With its non-invasive characteristic, real-time data collection capability, cost-effectiveness, and unique ability to capture rich 3D skeletal information, ReadyGo has quickly became an ideal choice for gait assessment in both clinical and scientific research. Through implicit monitoring, this technology not only reduced the discomfort of patients but also captured the most authentic gait data in a natural state, providing a solid foundation for precision medicine and personalized treatment.

Despite the increasing number of objective and quantitative assessments of gait disorder in PD patients in recent years, there is a relative lack of research focusing on the gait characteristics in the early stage of the disease. Our study aimed to deep explore the gait disorder characteristics of PD patients, especially those in the early stage, and to explore whether gait assessment can effectively identify differences between early-stage PD patients and healthy elderly individuals.

By conducting a detailed comparative analysis of gait parameters between early-stage PD patients and healthy controls (HC), we hoped to reveal the unique gait patterns of early-stage PD patients. This would not only help improve the accuracy and timeliness of early diagnosis but also provide key information for predicting PD progression and optimizing intervention strategies. Our research was expected to bring advancements to the early diagnosis and management of PD, especially in the aspects of identification and monitoring of gait disorder, opening up new methods for improving the quality of life for patients.

## 2 Materials and methods

### 2.1 Participants

In this study, 63 patients with primary PD were encompassed, with 27 being male and 36 being female. The inclusion criteria were as follows: (1) Meeting the 2015 Movement Disorder Society (MDS) diagnostic criteria for primary PD (14); (2) Hoehn and Yahr (H&Y) stages between stage 1 and 3; (3) The Mini-Mental State Examination (MMSE) score of 24 or above. Additionally, 65 gender- and age-matched healthy participants were selected as the healthy control group, including 35 males and 30 females. The ages of participants ranged from 46 to 85 years old, and all of them were able to complete the tests without any assistance from others. Exclusion criteria included: (1) Atypical Parkinsonism; (2) Severe systemic diseases (such as musculoskeletal, cardiovascular, cerebrovascular and respiratory) and other neurological diseases; (3) Uncorrected visual impairments, or diseases that could alter gait patterns. This study was approved by the Ethics Committee of Central Hospital of Dalian University of Technology (Reference No. YN2022-039-57). Each participant signed the informed consent before participating in this study. The study was performed according to the guidelines of the declaration of Helsinki.

### 2.2 Clinical assessment

Demographic information was collected, including age, gender, height (cm), weight (kg), and disease duration. All patients were assessed by two experienced neurologists in movement disorders. The severity of the disease were evaluated using the H&Y staging scale (15) which score ranged from 0 (no symptoms) to 5 (wheelchair bound or bedridden unless aided) and the MDS-UPDRS III (16) which consisted of 33 items with a score ranged from 0 (no symptoms) to 132 (severe motor symptoms). Cognitive was evaluated using the MMSE which score ranged from 0 to 30, with higher score indicating better cognitive function (17).

### 2.3 Gait assessment

**Figure 1:**
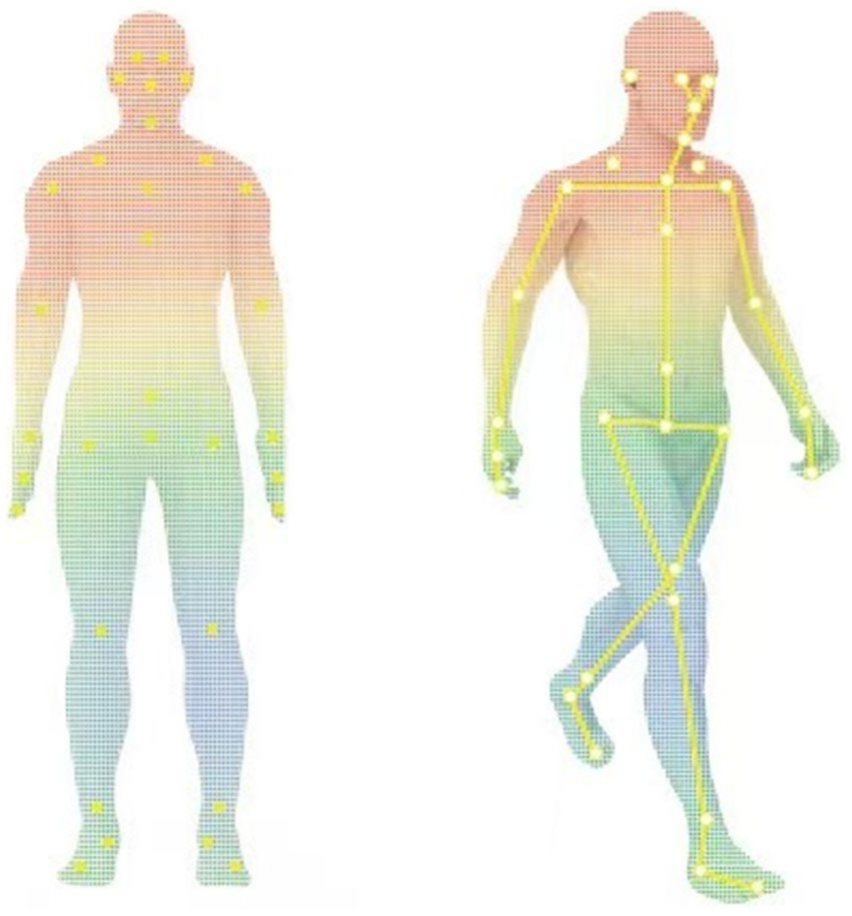
Human skeletal point tracking and motion recognition (the yellow markers indicate the key skeletal points tracked by the ReadyGo system).

Gait parameters were assessed using ReadyGo. Unlike the traditional multi-camera system, ReadyGo system innovatively utilizes a set of integrated cameras, including one RGB (red/green/blue) camera and a single depth camera, to capture and analyze three-dimensional (3D) motion data. The main advantage of this system lies in its unique skeletal tracking technology, which uses deep learning algorithms for precise positioning of skeletal points without requiring participants to wear any additional sensors, greatly enhancing the experience of the participants and the convenience of data collection (Figure 1). By meticulously analyzing the gait of PD patients, the system can automatically extract and quantify up to 19 key gait parameters, covering many aspects of the gait cycle, including gait speed, cadence, stride length, swing and stance phases, and so on, providing numerous details for a comprehensive understanding of gait disorder in PD patients. The accuracy and sensitivity of the ReadyGo system have been validated in previous studies (13), showing high reliability in capturing key gait parameters such as stride length and gait speed.

### 2.4 Procedure

**Figure 2:**
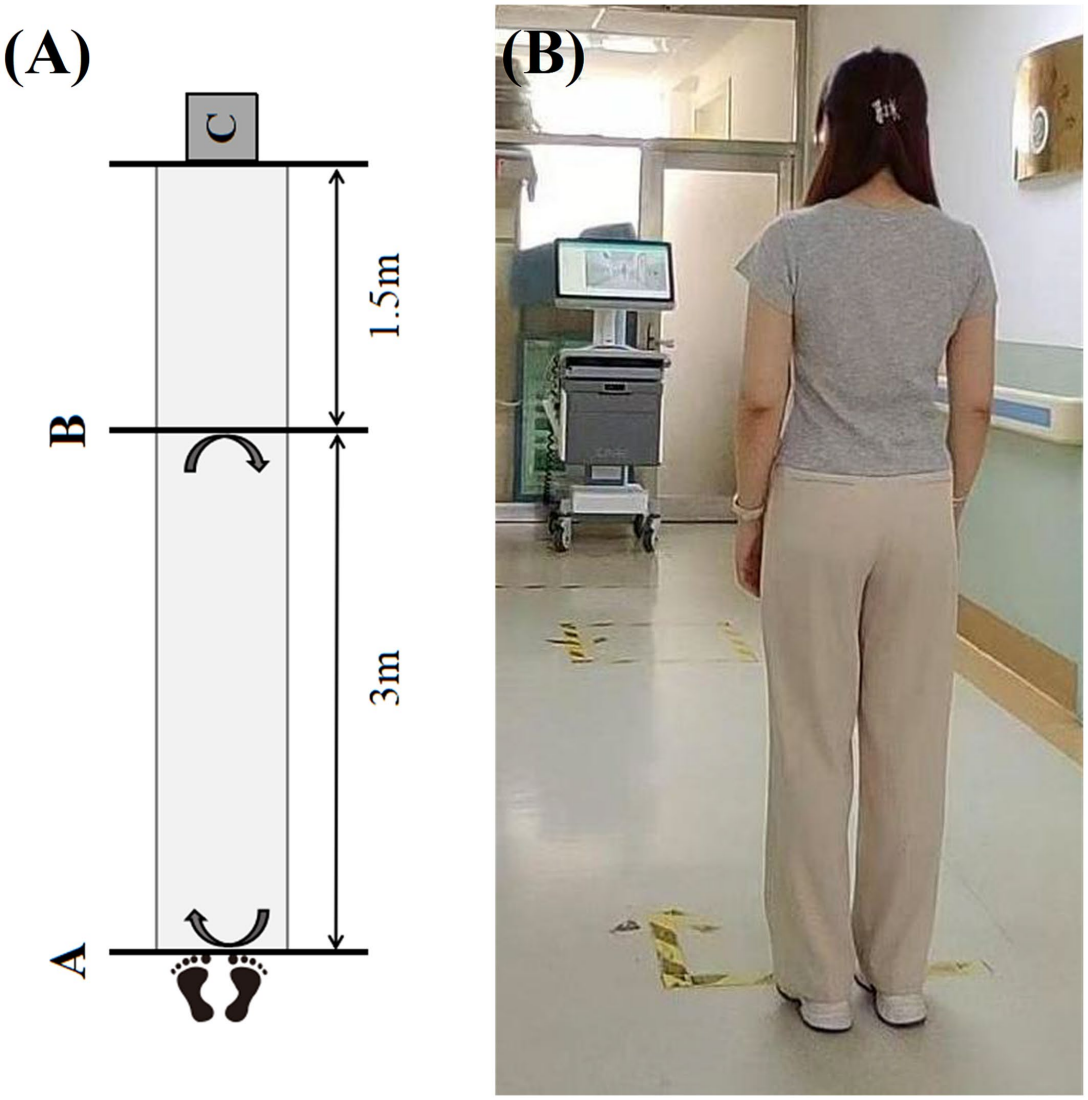
The procedure of gait assessment. **(A)** The schematic diagram of gait assessment (Line A is the starting line which is the departure point for the gait assessment; Line B is the end line which is the turning point for the gait assessment; C represents the area where the gait assessment device is placed). **(B)** The photograph of the actual procedure of gait assessment.

1. Scale assessment: MDS-UPDRS III and H&Y staging scale were performed by two experienced neurologists in movement disorders, and then gait assessment was carried out. PD patients underwent all of the above, and healthy controls only underwent gait assessment but without clinical scale assessments.
2. Gait assessment: the gait assessment device was placed in the equipment placement area which was 1.5 meters away from the endline. Participants stood at the starting line which was 4.5 meters directly in front of the device. During the test, participants were asked to walk at their self-selected comfortable pace without using any assistant device, start from the starting line, walk to the end line, turn around, and return to the starting line, repeat this process three times before ending the recording (Figure 2). Each participant should undergo a practice trial before the test to ensure that they understood the instructions clearly.

**Figure 3:**
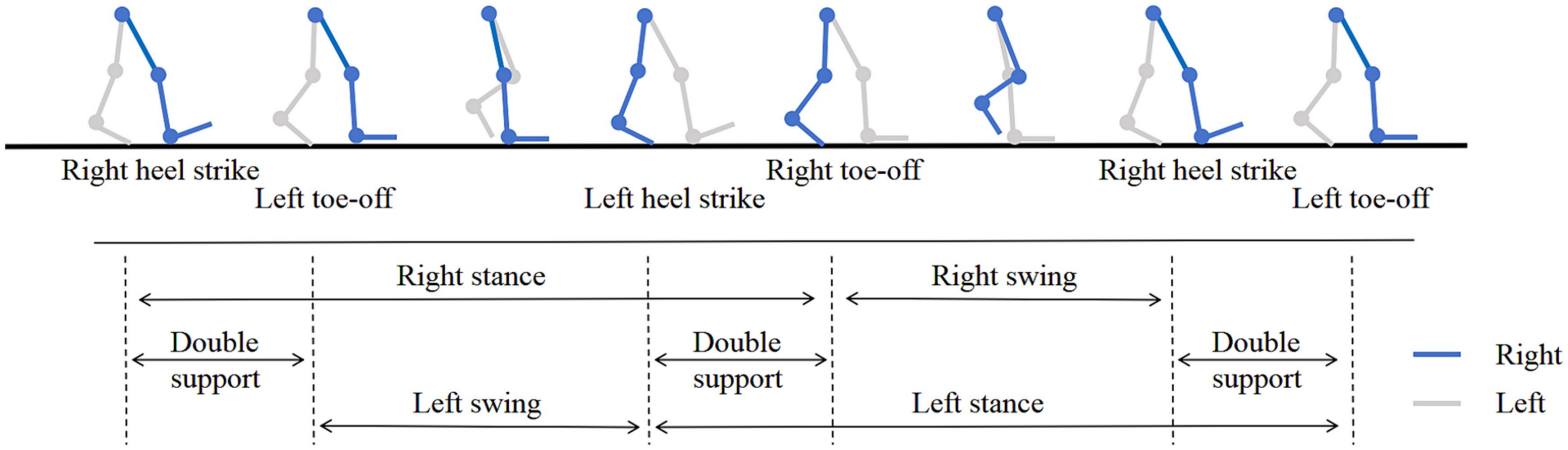
Gait cycle.

The gait data was achieved through a non-contact method, 19 gait parameters were extracted based on the images of gait and depth information, including stride length (left, right), step height (left, right), step width, gait speed, stride speed (left, right), swing speed (left, right), turning time, cadence (left, right), swing phase (left, right), stance phase (left, right), double support phase (left, right). The specific definitions were shown in Table 1 and the gait cycle was shown in Figure 3.

**Table 1:** Specific definitions of gait parameters in this study.

Gait parameter	Definition
Stride length-L/R (m)	The distance between two landings of the left/right foot.
Step height-L/R (m)	The highest distance from the ground during the swing of the left/right foot.
Step width (m)	The average of the width of the left and right feet in each image frame.
Gait speed (m/s)	Average speed during straight travel (not including the turning time).
Stride speed-L/R (m/s)	Average speed during a left/right stride.
Swing speed-L/R (m/s)	Average speed during a left/right swing.
Turning time (s)	The time from turning start to turning end.
Cadence-L/R (steps/min)	Frequency of left/right footstep.
Swing phase-L/R (%)	Percentage of left/right swing phase time in the left/right stride time.
Stance phase-L/R (%)	Percentage of left/right stance phase time in the left/right stride time.
Double support-L/R (%)	Percentage of double support phase time in the left/right stride time.

L, left; R, right.

### 2.5 Modeling

In this study, we defined patients in the H&Y 1 and 2 as early-stage PD and constructed a classification model using gait parameters of early-stage PD and healthy controls. The classification model was constructed to distinguish early-stage PD from healthy controls using gait parameters. Ten gait parameters with a statistical significance level of *p* < 0.001 were selected as input features. The dataset was split into a training set (70%) and a test set (30%) to evaluate the model’s performance. We utilized the LightGBM algorithm to build the classifier. The model was trained and validated using the training set, and its predictive performance was evaluated on the test set.

The Receiver Operating Characteristic (ROC) curve was performed as an important tool to evaluate the performance of the model, exploring whether gait parameters can identify early-stage PD from healthy controls. The ROC curve showed the relationship between the true positive rate (sensitivity) and the false positive rate (1-specificity), visually presented the classification capability of the model at different thresholds. In this study, we paid particular attention to the Area Under the Curve (AUC) of the ROC, the closer the value is to 1, indicated the better classification performance of the model, i.e., the stronger ability of gait parameters to identify early-stage PD.

By comprehensively analyzing the ROC curve and AUC value of the model, we hoped to validate the feasibility of gait parameters as early diagnostic biomarkers for PD, to provide a new perspective and basis for the early detection, intervention, and management of PD. This research would also lay a theoretical foundation for the subsequent development of more precise and personalized early screening tools for PD, promoting continuous advancement in clinical practice.

In the process of deepening our research, we adopted a more refined analytical strategy, using the Random Forest algorithm to train a machine learning regression model, which used all the 19 gait parameters as input features and MDS-UPDRS III score as output labels. This methodological shift aimed to explore how gait parameters quantitatively correlate with the severity of PD. By training a Random Forest regression model, we could predict the MDS-UPDRS III score of PD patients. To evaluate the performance of the model, we utilized the Leave-One-Out Cross Validation (LOOCV) method. In LOOCV, each instance of the dataset is used once as a test set while the remaining instances form the training set. This iterative process ensures that every data point is used for both training and validation, providing a robust assessment of the model’s generalization capability and predictive accuracy.

We could comprehensively evaluate the performance of the regression model by using these metrics: R-squared (R^2^), Mean Absolute Error (MAE), Mean Absolute Percentage Error (MAPE). R^2^ measured how well the model fitted the data, ranging from 0 to 1, the closer the value was to 1, indicated a better fit of the model. MAE quantified the average absolute difference between the predicted value and true value, the smaller value suggested higher predictive accuracy of the model. MAPE was the average absolute percentage error expressed as a percentage, which was better for assessing the relative error between predicted value and true value, which was often used to understand the prediction accuracy of a model over different ranges.

### 2.6 Statistical analysis

The statistical analysis in this study was analyzed using SPSS 26.0 (IBM Corp., Armonk, NY). Continuous variables with normal distribution were presented as mean ± standard deviation ( $\bar{x}$ ± *s*), the comparisons between two groups were made using the Independent Samples *t*-Test, the comparisons among multiple groups with homogeneous variances were made using one-way ANOVA followed by Bonferroni post-hoc test, the comparisons among multiple groups with non-homogeneous variances were made using the Welch test followed by Games-Howell (A) post-hoc test. Continuous variables with non-normal distribution were presented as median and interquartile distances [*M* (*P_25_*, *P_75_*)], and comparisons between two groups were made using the Mann–Whitney *U* test, while comparisons among multiple groups were made using the Kruskal-Wallis test followed by post-hoc test. Categorical variables were described by frequency, and group comparisons were made using the Pearson Chi-square test. The statistically significant difference was considered *p* < 0.05 in two-tailed tests. In this study, scikit-learn data analysis library of Python 3.8 was used to train and verify the classification and regression models. Meanwhile, drawing libraries such as matplotlib and seaborn were used to visually display the distribution characteristics of data and prediction effects of the models.

## 3 Results

### 3.1 Demographic and clinical characteristics

The demographic data between PD patients and the healthy controls were comparable (the first four lines in Table 2), there were no significant statistical differences in gender, age, height, and weight between the two groups (*p* > 0.05).

**Table 2:** Demographic and clinical characteristics.

Variable	PD (*n* = 63)	HC (*n* = 65)	*p* value
Gender (male/female)	27/36	35/30	0.214
Age (years)	66.90 ± 8.43	67.40 ± 7.17	0.721
Height (cm)	165.10 ± 7.98	165.46 ± 7.96	0.795
Weight (kg)	67.83 ± 12.38	66.92 ± 11.37	0.665
Disease duration (years)	1–10	NA	NA
MMSE (score)	24–30	NA	NA
H&Y stage		NA	NA
1	13		
2	38		
3	12		
MDS-UPDRS III (score)–overall	25.50 (17.00, 39.00)	NA	NA
MDS-UPDRS III (score)–H&Y 1	13.15 ± 4.30	NA	NA
MDS-UPDRS III (score)–H&Y 2	25.50 (21.00, 35.25)	NA	NA
MDS-UPDRS III (score)–H&Y 3	48.83 ± 11.89	NA	NA

PD, Parkinson’s disease; HC, healthy controls; MMSE, Mini-Mental State Examination; H&Y, Hoehn and Yahr; MDS-UPDRS III, section III of the modified movement disorder society version of the unified Parkinson’s disease rating scale; NA, not applicable.

The disease duration of PD patients ranged from 1 to 10 years, the MMSE score ranged from 24 to 30 points, the median MDS-UPDRS III score was 25.5 points, and H&Y stages ranged from 1 to 3, including 13 individuals in stage of H&Y 1, 38 individuals in stage of H&Y 2, and 12 individuals in stage of H&Y 3.

### 3.2 Comparison of gait parameters between PD patients and healthy controls

There were statistically significant differences in gait parameters between PD patients and healthy controls except for step width and right step height. The PD patients exhibited shorter stride length, slower gait speed, slower stride speed, slower swing speed, longer turning time, slower cadence, longer percentage of stance phase, shorter percentage of swing phase, and longer percentage of double support phase (Table 3). These results confirmed the “short and slow” gait characteristics of PD patients.

**Table 3:** Comparison of gait parameters between PD patients and healthy controls.

Gait parameter	PD (*n* = 63)	HC (*n* = 65)	*p* value
Stride length-L (m)	0.89 ± 0.23	1.10 ± 0.15	**< 0.001**
Stride length-R (m)	0.89 ± 0.24	1.09 ± 0.15	**< 0.001**
Step height-L (m)	0.10 ± 0.03	0.12 ± 0.02	**0.004**
Step height-R (m)	0.10 ± 0.03	0.10 (0.09, 0.12)	0.127
Step width (m)	0.14 (0.12, 0.15)	0.13 ± 0.02	0.091
Gait speed (m/s)	0.72 ± 0.23	1.10 (1.01, 1.21)	**< 0.001**
Stride speed-L (m/s)	0.80 ± 0.24	1.20 ± 0.21	**< 0.001**
Stride speed-R (m/s)	0.80 ± 0.24	1.20 ± 0.22	**< 0.001**
Swing speed-L (m/s)	1.93 ± 0.45	2.74 ± 0.36	**< 0.001**
Swing speed-R (m/s)	1.94 ± 0.47	2.75 ± 0.41	**< 0.001**
Turning time (s)	1.60 (1.21, 2.06)	1.03 (0.89, 1.31)	**< 0.001**
Cadence-L (steps/min)	112.49 (100.00, 119.99)	128.57 (120.00, 138.46)	**< 0.001**
Cadence-R (steps/min)	105.88 (100.00, 112.50)	128.57 (120.00, 141.76)	**< 0.001**
Swing phase-L (%)	31.17 ± 3.17	32.39 ± 2.69	**0.021**
Swing phase-R (%)	31.09 ± 3.50	33.33 (31.43, 34.62)	**< 0.001**
Stance phase-L (%)	68.82 ± 3.17	67.61 ± 2.69	**0.022**
Stance phase-R (%)	68.90 ± 3.50	66.67 (65.39, 68.57)	**< 0.001**
Double support-L (%)	38.14 ± 6.34	34.62 (33.33, 36.85)	**< 0.001**
Double support-R (%)	37.43 ± 6.21	34.62 (33.33, 36.85)	**0.004**

L, left; R, right. *p* values in bold indicate statistical significance (*p* < 0.05).

### 3.3 Comparison of gait parameters among H&Y 1, H&Y 2, and H&Y 3

**Figure 4:**
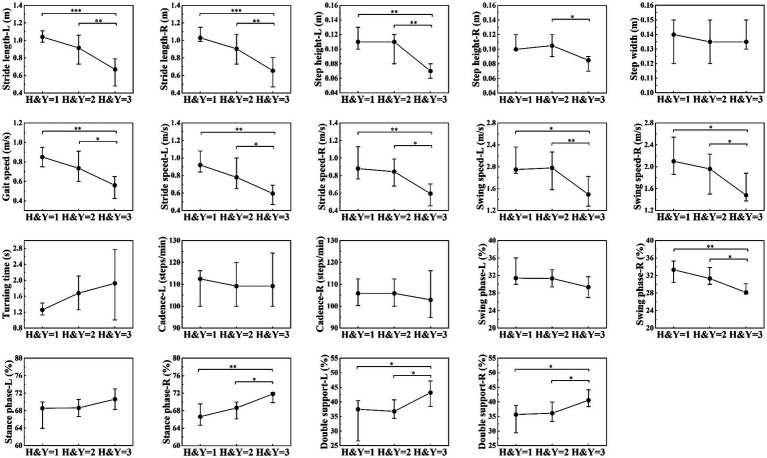
Comparison of gait parameters among H&Y 1, H&Y 2 and H&Y 3. The filled circles represent the median, lower and upper lines represent the 25th and 75th percentile, respectively. *represents *p* < 0.05, **represents *p* < 0.01, ***represents *p* < 0.001.

PD patients were divided into three groups according to the H&Y stages: H&Y 1, H&Y 2, and H&Y 3. We found that there were statistically significant differences in stride length, step height, gait speed, stride speed, swing speed, and percentage of double support phase among the three groups. Post-hoc test showed that these differences occurred between H&Y 1 and H&Y 3, and between H&Y 2 and H&Y 3, but there was no statistically significant difference when comparing H&Y 1 with H&Y 2. That was to say, compared with H&Y 1 and H&Y 2, H&Y 3 exhibited shorter stride length, lower step height, slower gait speed, slower stride speed, slower swing speed, and a longer percentage of time with both feet on the ground (Table 4; Figure 4).

**Table 4:** Comparison of gait parameters among H&Y 1, H&Y 2, and H&Y 3.

Gait Parameter	H&Y stage			*p* value			
	H&Y 1 (*n* = 13)	H&Y 2 (*n* = 38)	H&Y 3 (*n* = 12)	Overall	1 *vs* 2	1 *vs* 3	2 *vs* 3
Stride length-L (m)	1.03 ± 0.13	0.91 ± 0.22	0.66 ± 0.22	**<0.001**	0.060	**<0.001**	**0.007**
Stride length-R (m)	1.02 ± 0.17	0.91 ± 0.22	0.66 ± 0.23	**<0.001**	0.383	**< 0.001**	**0.001**
Step height-L (m)	0.11 ± 0.02	0.11 ± 0.03	0.07 ± 0.02	**<0.001**	1.000	**0.001**	**0.001**
Step height-R (m)	0.11 ± 0.03	0.11 ± 0.03	0.08 ± 0.02	**0.025**	1.000	0.068	**0.029**
Step width (m)	0.13 ± 0.02	0.13 ± 0.02	0.14 (0.13, 0.15)	0.706	-	-	-
Gait speed (m/s)	0.82 ± 0.16	0.75 ± 0.21	0.54 ± 0.24	**0.003**	0.855	**0.004**	**0.011**
Stride speed-L (m/s)	0.92 ± 0.19	0.82 ± 0.22	0.60 ± 0.23	**0.002**	0.420	**0.002**	**0.012**
Stride speed-R (m/s)	0.90 ± 0.22	0.83 ± 0.22	0.61 ± 0.24	**0.004**	0.984	**0.005**	**0.011**
Swing speed-L (m/s)	2.07 ± 0.32	1.99 ± 0.44	1.56 ± 0.44	**0.006**	1.000	**0.012**	**0.009**
Swing speed-R (m/s)	2.15 ± 0.42	1.97 ± 0.47	1.61 ± 0.40	**0.011**	0.686	**0.011**	**0.049**
Turning time (s)	1.36 ± 0.39	1.68 (1.26, 2.17)	1.95 ± 1.00	0.105	-	-	-
Cadence-L (steps/min)	107.28 ± 12.05	110.50 ± 13.05	114.74 ± 20.39	0.441	-	-	-
Cadence-R (steps/min)	106.83 ± 12.48	105.88 (99.34, 112.50)	107.87 ± 16.93	0.948	-	-	-
Swing phase-L (%)	32.31 ± 4.29	31.29 ± 2.57	29.55 ± 3.12	0.156	-	-	-
Swing phase-R (%)	33.19 ± 3.93	31.34 (29.91, 33.85)	28.51 ± 3.49	**0.005**	0.866	**0.006**	**0.021**
Stance phase-L (%)	67.68 ± 4.29	68.70 ± 2.57	70.45 ± 3.12	0.156	-	-	-
Stance phase-R (%)	66.80 ± 3.93	68.66 (66.14, 70.09)	71.49 ± 3.49	**0.005**	0.866	**0.006**	**0.021**
Double support-L (%)	34.96 ± 8.35	36.78 (34.24, 40.81)	43.10 ± 6.09	**0.012**	1.000	**0.018**	**0.027**
Double support-R (%)	34.91 ± 8.39	36.14 (33.33, 39.97)	41.79 ± 5.76	**0.014**	1.000	**0.018**	**0.037**

L, left; R, right. *p* values in bold indicate statistical significance (*p* < 0.05).

### 3.4 Comparison of gait parameters between early-stage PD and healthy controls

From Table 4, we could find that there was no statistically significant difference in gait parameters between H&Y 1 and H&Y 2. Therefore, we defined H&Y 1 and H&Y 2 as early stage and compared them with the healthy controls. We found that early-stage PD patients had shorter stride length (left, right), slower gait speed, slower stride speed (left, right), slower swing speed (left, right), slower cadence (left, right), and longer turning time. This indicated that the gait parameters such as stride length, gait speed, stride speed, swing speed, turning time, and cadence were the first to be affected in the early stage of PD.

### 3.5 ROC analysis

**Figure 5:**
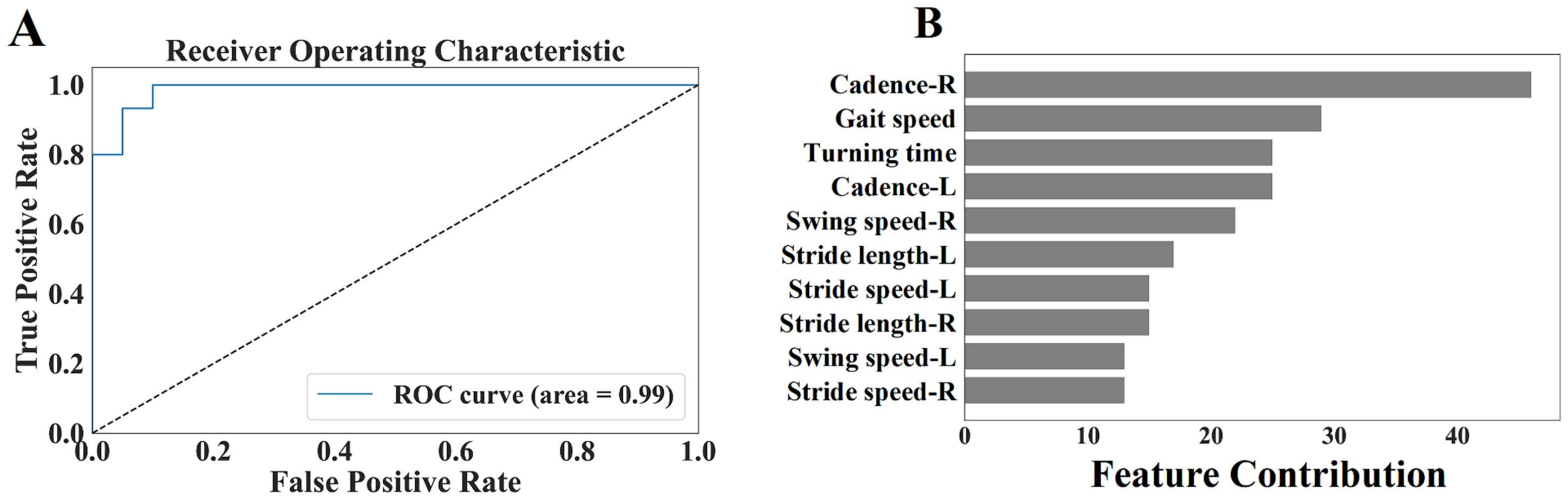
**(A)** Shows the ROC curve of the selected gait parameters in early-stage PD. **(B)** Shows the feature contribution of the selected gait parameters.

From Table 5, we selected 10 gait parameters with a statistical significance level of *p* < 0.001 when comparing early-stage PD with healthy controls, including stride length (left, right), gait speed, stride speed (left, right), swing speed (left, right), turning time, and cadence (left, right). We performed ROC analysis on the combined gait parameters mentioned above, and evaluated the ability of gait parameters to distinguish early-stage PD from healthy controls. The accuracy was 91.43%, the sensitivity was 93.33%, the specificity was 90.0%, and the area under the curve (AUC) was 0.99, indicating that the gait parameters could correctly distinguish 91.43% early-stage PD from healthy controls. We further analyzed the feature contribution of the included 10 gait parameters, feature contribution was evaluated using the SHAP (SHapley Additive exPlanations) method, which quantifies each parameter’s contribution to the model’s predictions. The contribution degree was in the following order: cadence (right), gait speed, turning time, cadence (left), swing speed (right), stride length (left), stride speed (left), stride length (right), swing speed (left), and stride speed (right). Cadence, gait speed, and turning time had the greatest influence of the gait parameters for distinguishing between early-stage PD and the healthy controls, while stride length, stride speed, and swing speed had a secondary influence (Figure 5).

**Table 5:** Comparison of gait parameters between early-stage PD and healthy controls.

Gait Parameter	Early-stage PD (*n* = 51)	HC (*n* = 65)	*p* value
Stride length-L (m)	0.94 ± 0.20	1.10 ± 0.15	**< 0.001**
Stride length-R (m)	0.94 ± 0.21	1.09 ± 0.15	**< 0.001**
Step height-L (m)	0.11 ± 0.03	0.12 ± 0.02	0.175
Step height-R (m)	0.11 ± 0.03	0.10 (0.09, 0.12)	0.781
Step width (m)	0.13 ± 0.02	0.13 ± 0.02	0.156
Gait speed (m/s)	0.76 ± 0.20	1.10 (1.01, 1.21)	**< 0.001**
Stride speed-L (m/s)	0.84 ± 0.22	1.20 ± 0.21	**< 0.001**
Stride speed-R (m/s)	0.85 ± 0.22	1.20 ± 0.22	**< 0.001**
Swing speed-L (m/s)	2.01 ± 0.41	2.74 ± 0.36	**< 0.001**
Swing speed-R (m/s)	2.02 ± 0.46	2.75 ± 0.41	**< 0.001**
Turning time (s)	1.56 (1.23, 2.05)	1.03 (0.89, 1.31)	**< 0.001**
Cadence-L (steps/min)	109.68 ± 12.77	128.57 (120.00, 138.46)	**< 0.001**
Cadence-R (steps/min)	105.88 (100.00, 112.50)	128.57 (120.00, 141.76)	**< 0.001**
Swing phase-L (%)	31.55 ± 3.08	32.39 ± 2.69	0.123
Swing phase-R (%)	31.42 (30.00, 33.97)	33.33 (31.43, 34.62)	**0.001**
Stance phase-L (%)	68.44 ± 3.08	67.61 ± 2.69	0.127
Stance phase-R (%)	68.57 (66.02, 70.00)	66.67 (65.39, 68.57)	**0.003**
Double support-L (%)	36.97 ± 5.86	34.62 (33.33, 36.85)	**0.008**
Double support-R (%)	36.41 ± 5.90	34.62 (33.33, 36.85)	0.086

L, left; R, right. *p* values in bold indicate statistical significance (*p* < 0.05).

### 3.6 Predicting MDS-UPDRS III score

**Figure 6:**
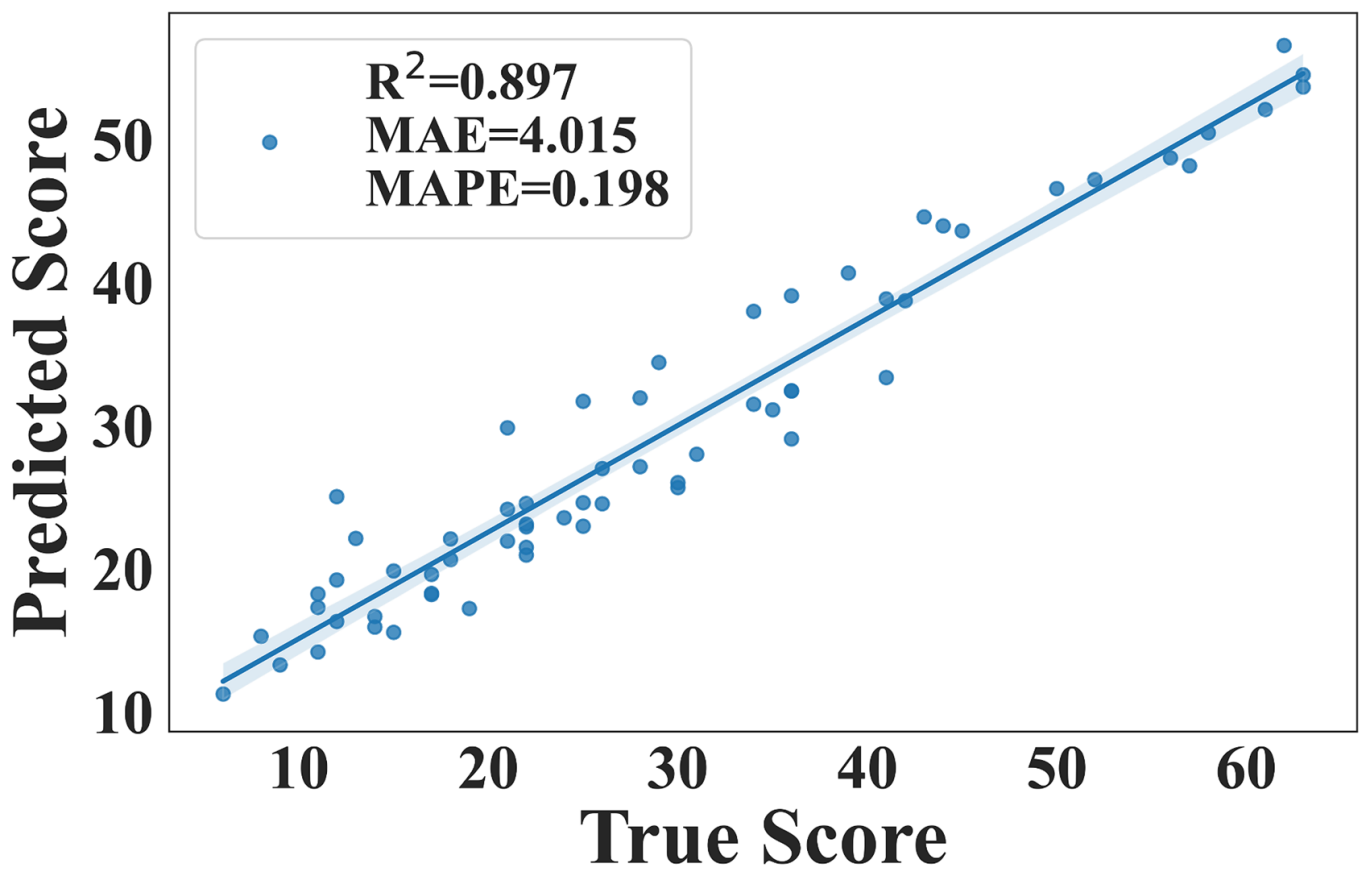
The scatter plot of the relationship between the predicted value and true value of the regression model (the horizontal axis of the scatter plot represents the true value of the MDS-UPDRS III score and the vertical axis represents the predicted value of the MDS-UPDRS III score).

To explore whether gait parameters could predict MDS-UPDRS III score, we trained a machine learning regression model using all the 19 gait parameters as input features and MDS-UPDRS III score as the output label. The scatter plot indicated that the model had a strong explanatory power for MDS-UPDRS III score (R^2^ = 0.897), with MAE of 4.015 and MAPE of 0.198 (Figure 6). The scatter plot showed the relationship between the predicted value and the true value of the regression model. It could be visually seen from the figure that the model performance for predicting MDS-UPDRS III score was good.

## 4 Discussion

Our study used a non-contact gait assessment system to assess and quantify gait parameters in patients with PD. Our findings confirmed that there were significant differences in gait parameters between PD patients and healthy controls. PD patients had slower gait speed and shorter stride length, which was consistent with the existing research. Gait is a very important motor function in daily life, gait disorder is closely associated with the quality of life (18). Therefore, early recognition and monitoring of gait is crucial for the diagnosis, treatment, and prognosis of PD patients. It is challenging to identify gait abnormalities in the early stage of PD, even to detect gait abnormalities without the complaint of gait disorder. Previous studies defined the early stage as H&Y stage below 2.5 (19, 20).

We divided PD patients into three groups according to H&Y stages and found that compared with H&Y 1–2, H&Y 3 had shorter stride length, lower step height, slower gait speed, slower stride speed, slower swing speed, and a longer percentage of time with both feet on the ground. But there was no statistically significant difference between H&Y 1 and H&Y 2. To explore gait abnormalities in the early stage of PD, we defined H&Y 1 and H&Y 2 as early stage, and compared them with healthy controls. We found that early-stage PD had shorter stride length, slower gait speed, slower stride speed, slower swing speed, slower turning time, and slower cadence. This indicated that in the early stage of PD, stride length, gait speed, stride speed, swing speed, turning time, and cadence were the first to be affected, suggested that they could be used to detect gait abnormalities in the early stage. In the progressive stage of the disease, step height, gait speed, stride speed, and swing speed decrease further, and the percentage of time with both feet on the ground was prolonged, indicated that these parameters could be used to monitor the progression of the disease.

We innovatively proposed an auxiliary diagnostic method based on fine-grained gait feature analysis, aimed to identify signs of PD in the early stage, we paid a particular attention to the potential value of gait parameters in the early diagnosis of PD. By using the machine learning model, we conducted an in-depth exploration of the selected ten gait parameters, including stride length (left, right), gait speed, stride speed (left, right), swing speed (left, right), turning time, and cadence (left, right). The results showed that these gait parameters could effectively distinguish early-stage PD from healthy controls. The model showed an encouraging classification performance, with an accuracy of up to 91%, the sensitivity of 93%, and the specificity also maintained at a high level of 90%. This strongly proved the practicality and reliability of the constructed model in the auxiliary diagnosis of early-stage PD.

To more comprehensively evaluate the potential of gait parameters in quantifying disease severity, we further explored their association with MDS-UPDRS III score. We constructed a predictive model using all the 19 gait parameters as input features and MDS-UPDRS III score as output label. The model showed excellent explanatory power and predictive accuracy: a high R^2^ value indicated that the model could effectively explain most of the score variations; a low MAE value indicated good consistency between the predicted value and true value of the model; and a low MAPE value highlighted the high precision of the model in predicting MDS-UPDRS III score. In summary, our research not only confirmed the importance of gait parameters in the early diagnosis of PD but also demonstrated their great potential in quantifying disease progression. This provided a new perspective and tool for the future clinical management and personalized treatment of PD.

While our study has revealed the potential of gait parameters in the auxiliary diagnosis of early-stage PD, there were still some limitations that should be acknowledged. The primary challenge lied in the limitation of sample size, that was, the relatively small number of participants. A small sample size might affect the power of statistical analysis and potentially constrain the generalizability and stability of the results. Additionally, the current study focused only on spatiotemporal parameters, such as stride length and gait speed, without involving more detailed kinematic parameters like joint angles. In light of these limitations, we plan to expand the sample size in future research and include more gait parameters, such as kinematic parameters, in order to build a more comprehensive and accurate diagnostic model.

## 5 Conclusion

In summary, the non-contact gait assessment system we used was capable of objectively and quantitatively evaluating gait disorder in PD patients, providing clinicians with a valuable tool for predicting MDS-UPDRS III score. Our machine learning models could accurately distinguish early-stage PD from healthy controls by integrating analysis of gait parameters such as stride length, gait speed, stride and swing speed, turning time, and cadence, and the model could also make reasonable prediction of MDS-UPDRS III score. This achievement reinforced the role of gait analysis in the early diagnosis of PD and paved the way for the development of early intervention and personalized treatment strategies for PD.

## Data Availability

The original contributions presented in the study are included in the article/supplementary material, further inquiries can be directed to the corresponding author.
